# Thoracic Wall Reconstruction after Tumor Resection

**DOI:** 10.3389/fonc.2015.00247

**Published:** 2015-10-29

**Authors:** Kamran Harati, Jonas Kolbenschlag, Björn Behr, Ole Goertz, Tobias Hirsch, Nicolai Kapalschinski, Andrej Ring, Marcus Lehnhardt, Adrien Daigeler

**Affiliations:** ^1^Department of Plastic Surgery, Burn Center, Hand Center, Sarcoma Reference Center, BG-University Hospital Bergmannsheil Bochum, Bochum, Germany

**Keywords:** thoracic wall, chest wall, tumor, sarcoma, breast cancer, reconstruction, flaps

## Abstract

**Introduction:**

Surgical treatment of malignant thoracic wall tumors represents a formidable challenge. In particular, locally advanced tumors that have already infiltrated critical anatomic structures are associated with a high surgical morbidity and can result in full-thickness defects of the thoracic wall. Plastic surgery can reduce this surgical morbidity by reconstructing the thoracic wall through various tissue transfer techniques. Sufficient soft-tissue reconstruction of the thoracic wall improves quality of life and mitigates functional impairment after extensive resection. The aim of this article is to illustrate the various plastic surgery treatment options in the multimodal therapy of patients with malignant thoracic wall tumors.

**Materials and methods:**

This article is based on a review of the current literature and the evaluation of a patient database.

**Results:**

Several plastic surgical treatment options can be implemented in the curative and palliative therapy of patients with malignant solid tumors of the chest wall. Large soft-tissue defects after tumor resection can be covered by local, pedicled, or free flaps. In cases of large full-thickness defects, flaps can be combined with polypropylene mesh to improve chest wall stability and to maintain pulmonary function. The success of modern medicine has resulted in an increasing number of patients with prolonged survival suffering from locally advanced tumors that can be painful, malodorous, or prone to bleeding. Resection of these tumors followed by thoracic wall reconstruction with viable tissue can substantially enhance the quality of life of these patients.

**Discussion:**

In curative treatment regimens, chest wall reconstruction enables complete resection of locally advanced tumors and subsequent adjuvant radiotherapy. In palliative disease treatment, plastic surgical techniques of thoracic wall reconstruction provide palliation of tumor-associated morbidity and can therefore improve patients’ quality of life.

## Introduction

The majority of thoracic wall defects result from the surgical resection of malignant tumors during curative or palliative attempts. These malignant tumors arise from all different anatomic structures of the thoracic wall and consequently vary in pathology and prognosis. Solid malignancies of the thoracic wall include primary thoracic wall tumors and metastatic lesions as well as locally invading malignancies from adjacent tissues and organs, such as breast cancer, lung cancer, mediastinal neoplasms, and mesothelioma. The most common primary thoracic wall tumors are bone and soft-tissue sarcomas. Approximately 55% of the primary malignant chest wall tumors arise from the bone or cartilage, whereas 45% originate from the soft tissue (1, 2). Chondrosarcomas are the most common skeletal malignancies of the thoracic wall and commonly occur in the anterior thoracic wall (3). In the heterogeneous group of soft-tissue sarcomas, not otherwise specified sarcomas (NOS) and liposarcomas are known to be the most frequent primary soft-tissue sarcomas of the thoracic wall (4). Notably, the incidence of radiation-induced angiosarcomas of the chest wall is increasing due to the prolonged survival of women irradiated for primary breast cancer and will present a therapeutic challenge in the future (5). In patients with primary chest wall tumors and radiation-induced angiosarcomas, nearly all treatment regimens involve the surgical resection of the tumor with clear margins, usually followed by adjuvant radiation and/or chemotherapy depending on the histologic entity. However, surgical resection and reconstruction of the thoracic wall are also suitable for other patients besides those with primary tumors. Increasing knowledge in all fields of modern medicine and effective treatment modalities for different types of cancer continuously increase the survival of patients with metastatic or locally advanced disease stage. The incidence of metastatic lesions of the chest wall and locally invading tumors from the breast and lung will become more frequent in the future. Thus, palliative treatment options with as little perioperative morbidity as possible will become increasingly important. In this palliative setting, resection of painful, odor-intensive, and bleeding tumors with subsequent thoracic wall reconstruction seems to be a valid option to increase the quality of life at least for a period of time. Hence, careful planning and individualized treatment are particularly important in these patients to provide a safe and fast recovery.

Nevertheless, partial- and full-thickness thoracic wall resections combined with reconstruction still represent a formidable surgical challenge, but improvements in surgical technique, intensive care, and rehabilitation have led to reduced perioperative morbidity and mortality (6, 7). In the surgical field, plastic surgery procedures enable oncologic tumor resection, reconstruction of the thoracic wall, and adjuvant radiotherapy by improving the local tissue situation (8). Moreover, plastic surgical reconstruction of the thoracic wall provides sufficient stability to maintain pulmonary function. Pulmonary function parameters are reduced only moderately and are not significantly affected by the size of the resection or its location (9).

In the following article, we will discuss the different options for thoracic wall reconstruction after oncological resection by examining a series of cases from our institution and a review of the literature.

## Preoperative Evaluation

Preoperative evaluation should be performed properly and in a multidisciplinary manner with pulmonary and cardiac function tests. In particular, patients with chronic obstructive pulmonary disease should be treated preoperatively to optimize pulmonary function before surgery. Postoperatively, patients with cardiac or pulmonary disorders should be treated in the intensive care unit, and early extubation and active respiratory therapy should be the most important treatment goals. Chest X-ray, CT, and MRI can be used as diagnostic tools to assess the imaging appearance of thoracic wall tumors. CT can provide additional information about calcification, bone destruction, and vascularity of the tumor, whereas MRI provides more soft-tissue details. Precise radiological examination with detailed information about tumor location and extent is essential for proper surgical planning and management as well as preoperative histologic evaluation. CT-controlled biopsy and incisional biopsy can be used as suitable modalities of tissue obtainment. Preoperative histologic examination is mandatory and should be performed in any lesions suspected to be malignant.

## Resection

In a curative setting, the aim of surgical treatment is the resection of the tumor with microscopically negative margins. Appropriate oncologic resection should not be compromised because of concern for the resulting thoracic wall defect. However, the extent of surgical margin width is determined by the chest wall tumor histology. In soft-tissue sarcomas, there has been a shift of the paradigms regarding the width of surgical resection from radical wide resections to more marginal resections (10–12). In the surgical treatment of primary soft-tissue sarcomas of the chest wall, negative surgical margins were not significantly associated with prolonged overall survival when compared with positive margins (7, 13). However, the attainment of microscopically negative margins should be the goal of surgical resection to improve local control and to prevent local recurrence (14), but, to date, there is no reasonable evidence for radical surgical approach in most soft-tissue sarcomas, for which marginal resections seem to be sufficient for local disease control (12).

Complete surgical resection with negative margins also remains the mainstay of therapy in the curative treatment of other malignancies that are still localized and not disseminated, such as locally advanced breast carcinomas. Thoracic wall resection and reconstruction have been proven to be a safe and effective procedure in patients with advanced, locally recurrent breast carcinomas (15).

As mentioned earlier, increasing numbers of patients present with a disseminated disease stage and are not suitable for a curative approach. In these patients, surgical treatment should be considered carefully and every attempt should be made to minimize perioperative morbidity. Tumor debulking and reliable soft-tissue coverage can alleviate pain and suffering for at least a period of time.

## Thoracic Wall Reconstruction with Mesh and Composite Implants

Depending on the extent of the malignant tumor, adequate oncologic resection can result in partial- or full-thickness thoracic wall defects. Full-thickness defects, which involve all tissue layers including soft tissue and bony structures, should be reconstructed immediately during the same surgery to protect the subjacent organs and to enable quick recovery. In this procedure, thoracic wall reconstruction should obliterate dead space and provide adequate soft-tissue coverage and stability, without compromising respiratory biomechanics. For this purpose, synthetic nets can be utilized to improve chest wall stability and to avoid herniation of intrathoracic organs. These nets should be both robust and pliable. In recent decades, synthetic nets have included essential features such as inertness, radiolucency, sufficient rigidity, and pliability. At our institution, we have had successful experiences with non-absorbable polypropylene meshes. However, different synthetic nets are now available, but none of them have proven to be significantly superior (16–18). The decision as to whether synthetic nets should be utilized depends on several factors, which include not only defect area and depth but also rigidity of the chosen flap coverage, location, wound contamination, and skin texture after previous radiation. It is widely accepted that defects exceeding more than four ribs at the lateral chest wall are associated with higher risks of herniation and paradox breathing and therefore should additionally be reconstructed with synthetic nets (8, 19–22). However, the closer the defect to the apex of the thoracic wall, the more suspension is provided by the sternum, scapula, and clavicula, and even larger defects might be reconstructed without additional synthetic material (23). Similarly, an irradiated chest wall may provide enough rigidity to avoid additional mesh implantation. Nevertheless, irradiated tissue should be replaced as far as possible by healthy tissue to allow proper healing and, if necessary, subsequent radiation (24). Notably, synthetic nets should be avoided in contaminated wound defects and should be implanted subsequently under clean wound conditions. Alternatively, if quick coverage and adequate stability can be achieved during the same surgical procedure, chest wall reconstruction can be performed with a stable, muscular flap, such as the latissimus dorsi flap, which is discussed below. In patients with simultaneous irradiated soft-tissue defects and infections in the chest wall area, such as pleural empyema, we usually debride and cover the defects with pedicled latissimus dorsi flaps without synthetic mesh implantation during one surgical procedure.

To maintain chest wall rigidity and to improve functional as well as cosmetic outcomes after large anterior and lateral resections, several authors have recommended the use of composite implant techniques (16, 21, 25–28). The most common composite is the combination of polypropylene meshes and methylmethacrylate substitutes in the form of a “sandwich” prosthesis. Here, a first layer of polypropylene mesh is positioned straight on the base of the defect and the methylmethacrylate substitute is then added and molded to the pattern of the defect. A second layer of polypropylene mesh is placed on top of the methylmethacrylate substitute, which hardens in an exothermic reaction. This composite implant technique allows for the reconstruction of the original contours of the chest wall and can be performed as a one-stage surgical procedure for major anterior and lateral chest wall defects to prevent paradoxical movement and overcome deformities. However, methylmethacrylate substitutes are not permeable to fluids and, hence, are considered to increase the risk of infections (29). Nevertheless, several case series and a retrospective analysis of 112 patients with polypropylene mesh/methylmethacrylate composites have demonstrated quite good functional results without increased infection rates (16, 26, 28). Weyant and colleagues have reported no significant difference between large chest wall defects reconstructed with polypropylene mesh/methylmethacrylate composite and small chest wall defects reconstructed with polypropylene mesh with regard to respiratory complications (28). Other composite implant techniques, including silicone, rubber, carbon fiber, and polytetrafluoroethylene (PTFE), have been described in case reports (21, 29–31). There have also been reports on the safe use of titanium implants in the reconstruction of the chest wall after tumor resection (32–34). In 19 patients with large anterior and lateral full-thickness defects after tumor resection, Berthet et al. have reconstructed the chest wall via a combination of titanium rib osteosynthesis and PTFE mesh in a one-step procedure (32). There were two cases of infection and one patient with a major complication in the form of respiratory failure. More recently, the improvements in 3D prototyping technology by selective laser sintering have enabled the production of more complex and detailed custom-made titanium implants. In this regard, Turna et al. have presented a case in which an extended anterior chest wall defect after tumor resection was safely reconstructed with a customized titanium implant in combination with a pedicled latissimus dorsi flap and a split-thickness graft (34). However, each material has its own advantages and disadvantages. There is still a lack of evidence regarding each of these approaches, and further studies are warranted to provide long-term data. The same issue applies to the use of allografts and xenografts because the literature on these topics remains sparse (21). The decision about which material to use ultimately depends on the defect and the surgeon’s experience.

## Osteosynthesis

When direct approximation of the sternal edges is possible, osteosynthetic procedures can maintain the chest wall stability and improve the functional outcome after partial anterior resections. Here, several studies have demonstrated that primary sternal plating reduces the risk of sternal non-unions and postoperative mediastinitis more effectively than does fixation via cerclage wires (35–37). If direct sternal fixation is not possible, we bridge over the sternal defect with local flaps such as the pectoralis major or vertical rectus abdominis muscle (VRAM) flap.

In the following section, we will address the different options of plastic surgical soft-tissue coverage that are commonly used at our institution.

## Thoracoepigastric Flap

**Figure 1:**
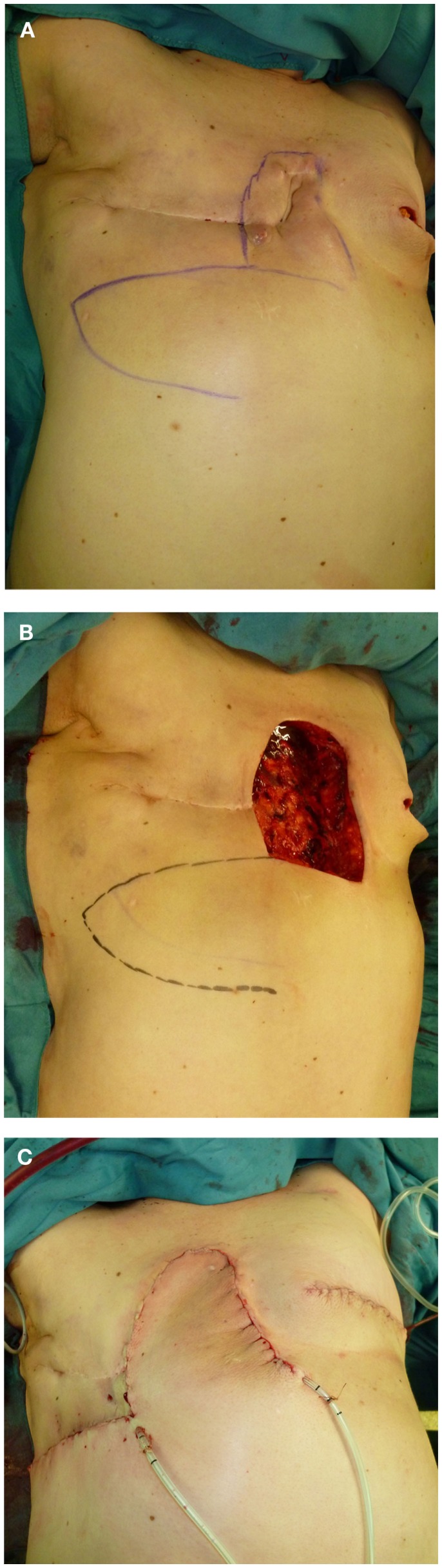
**(A–C)** Thoracoepigastric soft-tissue coverage after resection of a locally recurrent breast carcinoma (right) with simultaneous, contralateral infestation at the left breast.

The thoracoepigastric flap is a fasciocutaneous flap pedicled to the perforators at the proximity of the midline of the fascia of the musculus rectus abdominis and can be utilized to cover smaller defects (Figures 1A–C). Medially based thoracoepigastric flaps receive perforator vessels from the epigastric arcade, whereas laterally based flaps are supplied by perforators from the intercostal arteries. The reliability of the blood supply can be assessed by preoperative Doppler imaging. Thoracoepigastric flaps can be raised superior or inferior to the level of the rectus fascia and investing fascia of the external oblique musculature (38). Primary donor-site closure can be achieved for most of the laterally based flaps, whereas skin grafting is often required for medially based thoracoepigastric flaps (38, 39). Thoracoepigastric flaps are indicated for the coverage of smaller defects located in the lower thoracic region.

## Pectoralis Major Flap

**Figure 2:**
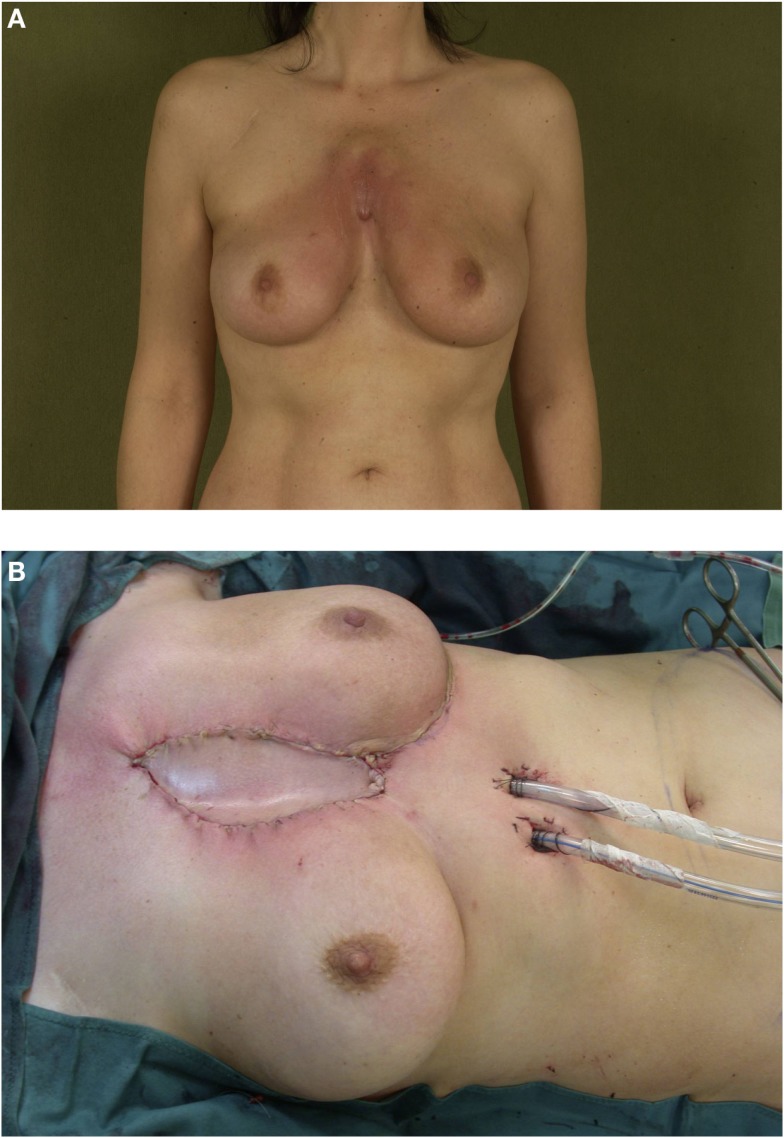
**(A,B)** Pectoralis major flap coverage of a central chest wall defect after resection of a recurrent sarcoma.

**Figure 3:**
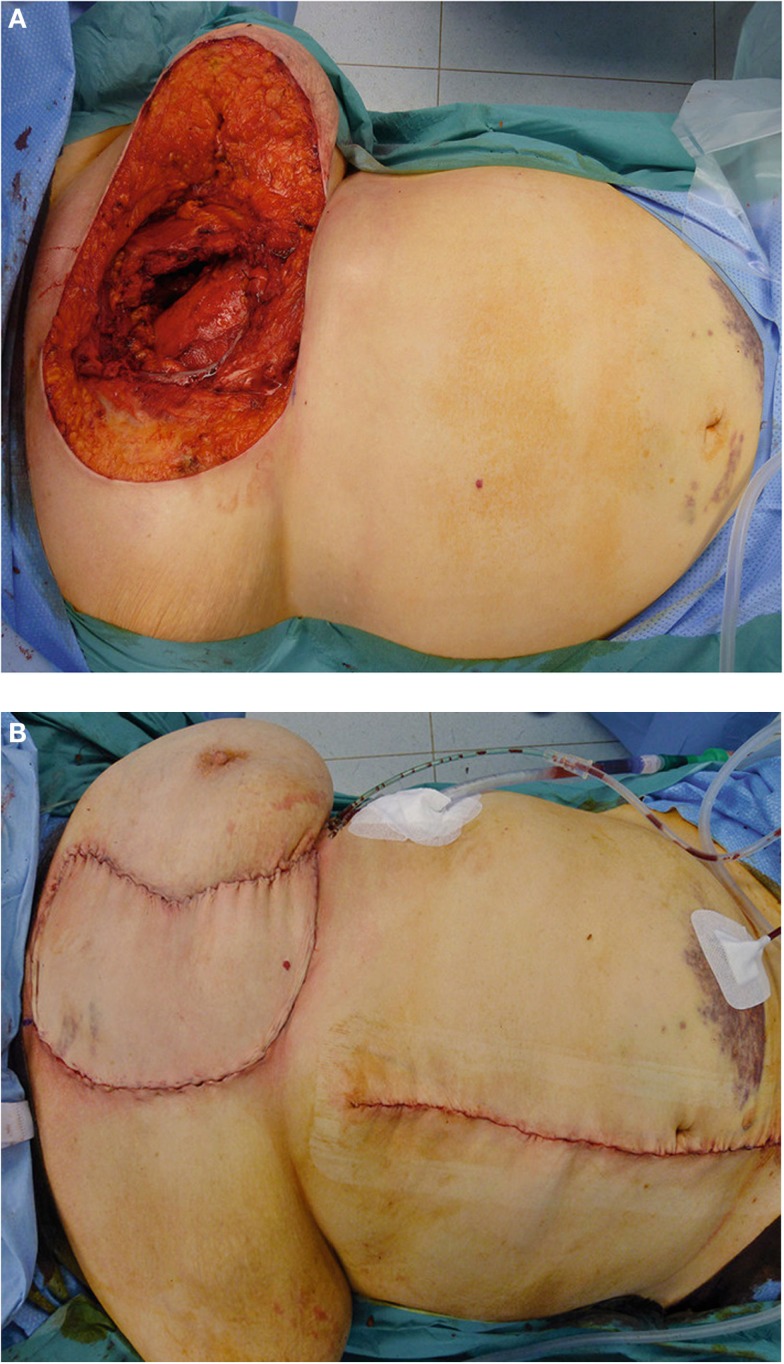
**(A,B)** Chest wall coverage following tumor resection with VRAM flap.

The pectoralis major flap can be used as a myocutaneous flap or simply as a muscular flap. When used as a myocutaneous flap, a skin graft is also taken from the region of the lower breast fold, and this graft remains pedicled to the muscle and can be transposed into the head and neck region (40). The pectoralis major muscle is supplied by a dominant vascular pedicle (arteria thoracoacromialis) and several minor pedicles. The thoracoacromial artery presents a consistent and reliable pedicle on which the pectoralis major muscle can be completely elevated (41). The pectoralis major muscle has also reliable secondary perforators from the internal mammary artery allowing medially based propeller flaps to cover smaller sternal defects. In chest wall reconstruction, the pectoralis major flap is primarily used as a muscle advancement or rotation flap to cover defects in the cranial portion of the sternum (42). Smaller contralateral defects may also be easily reached by this flap (Figures 2A,B and 3A,B). It can also be lifted from the thoracic wall as a sliding pectoralis muscle flap. To gain more rotatory flexibility, it can be removed from the clavicle and the humerus. In this case, it remains pedicled to the pectoral branches of the thoracoacromial artery. Upon lifting the muscle, there is only a moderate loss of strength (42). However, the size of the skin graft is very limited when lifted as a myocutaneous flap, and the vascular structure of the flap is often impaired by prior operations and radiotherapy. Low sternal and xiphoid defects may also be out of reach for the pectoralis major flap.

## VRAM Flap

**Figure 4:**
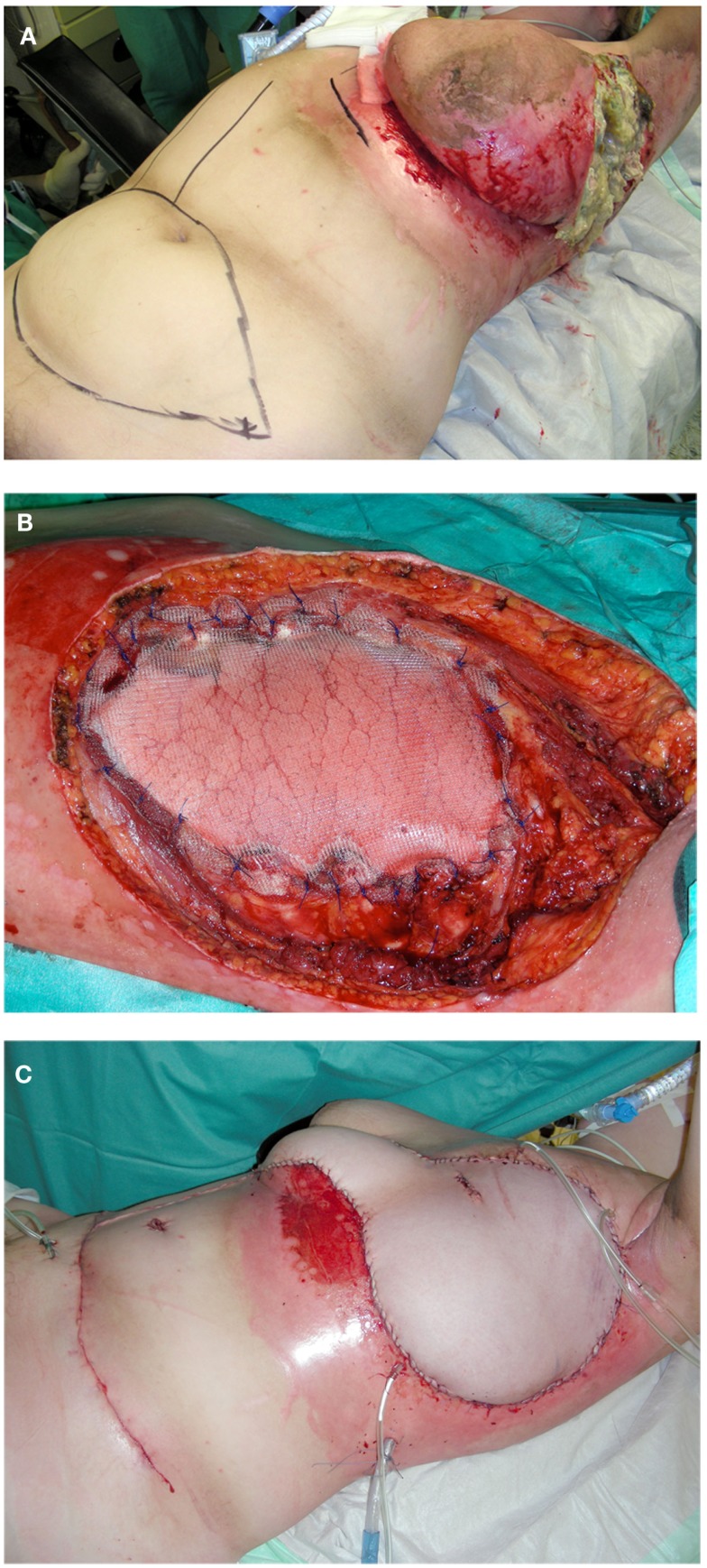
**(A–C)** Resection of ulcerative breast carcinoma and subsequent chest wall reconstruction with implantation of polypropylene mesh and cranially pedicled TRAM flap plasty.

The VRAM flap is particularly suited for longitudinal anterior chest wall defects (43) (Figures 4A,B). Preoperative planning should consider any possible removal of the arteria mammaria interna in previous coronary artery bypass operations because the VRAM flap is primarily supplied by the arteria epigastrica superior and arteria mammaria interna. In such cases, the VRAM flap can be lifted contralaterally to the place of removal. On rare occasions, insufficient venous outflow via the superior epigastric vessels can occur. Here, the inferior epigastric vessels at the caudal portion of the flap can be connected parasternally to the mammaria interna vessels in the sense of vessel supercharging. The VRAM flap is particularly indicated when sternal defects with large volumes should be covered and when sternal defects extend inferiorly to the epigastric areas (41, 44). It is also a reliable backup option when defect coverage with the latissimus dorsi flap is not possible. In a follow-up study at our institution, abdominal hernia and bulging occurred in 13% of all oncological patients treated with VRAM flap plasties. No flap loss was observed, and the loss of strength was moderate with a slight restriction of endurance without decreased maximum strength (45). However, the relatively high rates of abdominal hernia have to be considered, and the indication for local reconstruction with VRAM should be weighed carefully, especially in patients in a palliative setting where some surgical procedures (e.g., stabilization of the abdominal wall) should be avoided.

## Cranially Pedicled TRAM Flap

**Figure 5:**
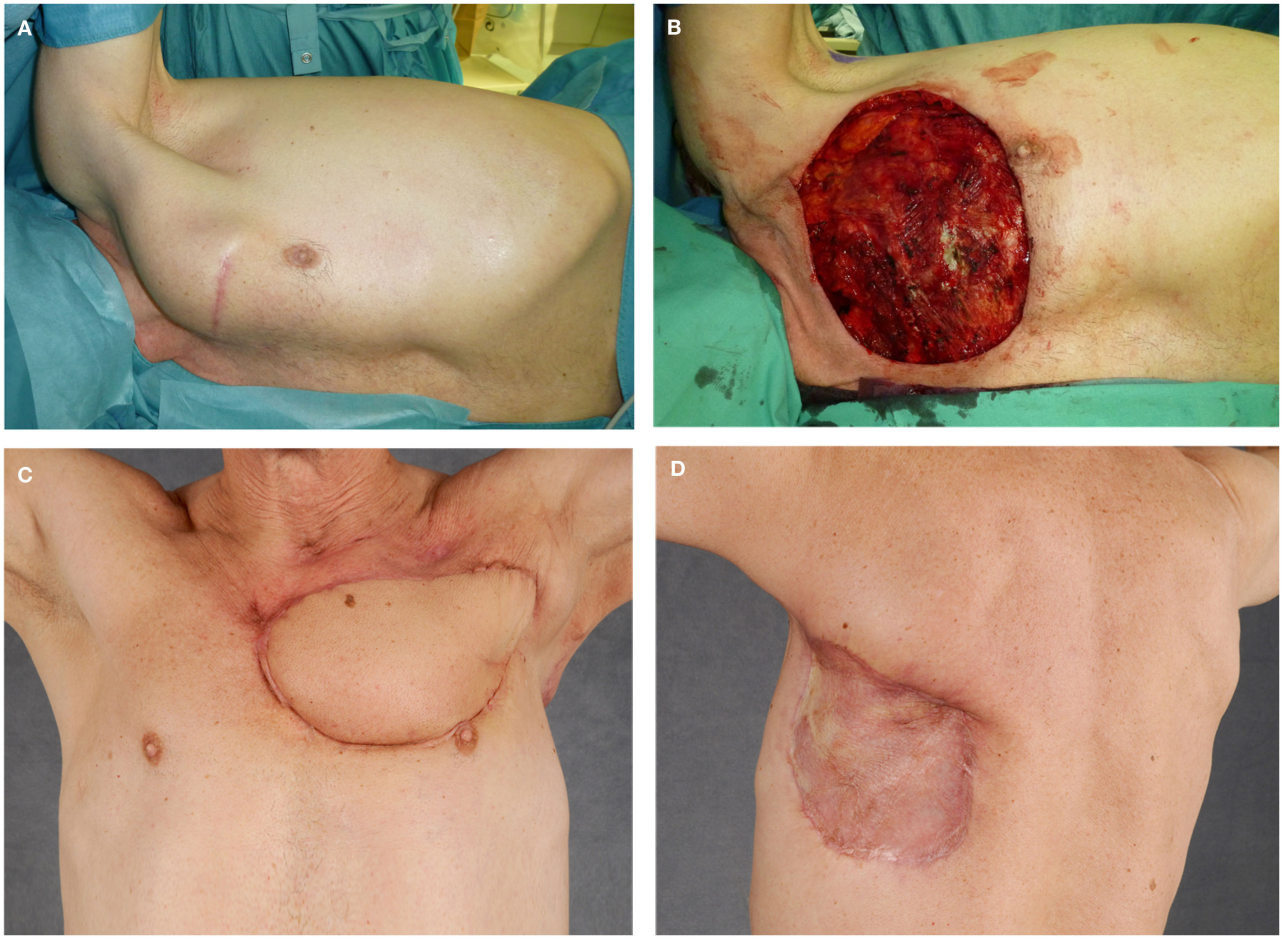
**(A–D)** Resection of a recurrent high-grade chondrosarcoma and chest wall reconstruction utilizing latissimus dorsi flap with split skin graft coverage of the flap donor site.

To cover larger defects, particularly at the anterolateral thorax, the VRAM flap can be extended to include a transversal graft from the lower abdomen [transverse rectus abdominis myocutaneous flap (TRAM)] (Figures 4C and 5A,B). The resulting anchor flap can correct defects up to 40 cm in diameter. In the majority of cases, the cutaneous donor site should primarily be closed by means of an abdominoplasty with umbilical repositioning when possible. Depending on the resulting fascia defect, the abdominal wall can be reinforced with a polypropylene mesh insert to avoid the formation of an abdominal hernia. The perfusion of cranially pedicled TRAM flap takes place via the superior epigastric vessels, which are slimmer than the inferior epigastric vessels. Hence, in the case of a cranially pedicled flap from the lower abdomen, perfusion disorders and partial necrosis can occur, particularly in the lateral portions of the flap.

Nevertheless, the cranially pedicled TRAM flap remains a reliable option in the armamentarium of soft-tissue coverage, especially in the case of extensive tumors of the anterolateral chest wall.

## Latissimus Dorsi Muscular Flap

A pedicled latissimus dorsi flap can sufficiently cover most defects on the thoracic wall (Figures 5C,D). The latissimus dorsi flap can be harvested as a muscle flap, a myocutaneous flap, or a perforator flap. The thoracodorsal artery is the dominant pedicle of the latissimus dorsi flap and arises from the subscapular artery. Anatomic variations are well described and should be considered when raising the flap (41). After entering the base of the latissimus dorsi muscle, the thoracodorsal artery divides into two main branches. The upper horizontal branch runs medially along the superior border of the muscle and the descending branch runs parallel to the anterior border of the muscle (46, 47). The large radius of rotation enables large soft-tissue coverage at the anterior chest wall, the sternal region, and the upper arm. Due to its reliable vascular supply, its proportions, and the moderate donor-site defect, the latissimus dorsi flap has proven itself in the coverage of thoracic wall defects (9, 20, 44, 48, 49). Because of its volume, it can also seal intrathoracic defects and dead space. The large caliber of the vascular pedicle with a diameter of 2–4 mm will also permit immediate microsurgical transfer if necessary (50).

## Omentum Majus Flap

The omentum majus flap is an alternative option for closing defects in the anterior thoracic wall when the aforementioned pedicled flaps or free flaps flap are not suitable. It is also an option to cover large defects with small volumes. Pedicled to the unilateral or bilateral gastro-omental vessels, it can be lifted via a paramedian incision from the xiphoid process to beneath the umbilicus (51, 52). The size of the omentum majus flap can only be determined reliably under direct visual control after surgical exposition. Especially after previous abdominal surgery, adhesions must be removed and the omentum majus raised from the stomach to achieve the appropriate rotatory radius. Furthermore, a breach must remain in the cranial abdominal wall so that the pedicle can be guided through it toward the thoracic wall. Consequently, this flap should be raised only by experienced surgeons who can manage potential intra-abdominal complications such as intestinal perforations and bleeding. Due to its great plasticity, the omentum is well suited for sealing dead space. However, it must always be covered by split skin graft and partial secondary healing can occur due to persistent serous discharge from the fatty tissue. Because of the high risk for the development of epigastric hernia and the aforementioned disadvantages, the pedicled omentum majus remains principally a fallback option when other procedures are not suitable (53, 54).

## Free Flap Plasties

Previous operations, axillary lymph dissection, or radiotherapy can prevent pedicled flaps from being utilized for soft-tissue coverage. In these situations, free flaps form an additional and pivotal tool in the armamentarium of plastic surgery. Fasciocutaneous or myocutaneous flaps from the back (latissimus dorsi flap, parascapular flap) or the thigh [anterior lateral thigh (ALT); tensor fasciae latae (TFL)] are some free flaps regularly used at our institution. Another frequently used donor area is the abdominal region with the TRAM flap or its muscle-preserving variation (ms-TRAM) as well as the perforator-based deep inferior epigastric artery perforator flap (DIEP).

The internal mammary artery is the primary connecting vessel at the anterior thoracic wall. At the lateral thoracic wall, the thoracodorsal vessels can act as sufficient connectors. In the event that these are not available, an arteriovenous loop between the cephalic vein and the thoracoacromial artery can constitute an effort-intensive but feasible solution (55).

The donor-site morbidity of free flaps is moderate and well tolerated by most patients, especially if the donor site can be closed primarily (50, 56, 57).

## Pulmonary Function, Quality of Life, and Mortality

In our patient population, thoracic wall reconstruction-impaired pulmonary function parameters vary only slightly (9). The most significant alteration was found in the forced expiratory volume in 1 s (FEV1), which was decreased by approximately 18%. This observed reduction of FEV1 might be the consequence of the loss of the intercostal muscles. However, the extent of chest wall resection was not found to be a significant predictor of pulmonary function alteration. In fact, breathing pain affected respiratory function in a significant manner, whereas the extent of resection surprisingly did not correlate with breathing pain. Partial lung resection also did not significantly impair pulmonary function. Similar findings were also observed in other studies in which pulmonary function was only slightly affected by thoracic wall resection (58–60). Reviewing our own data, hospitalization at our institution averaged 20.7 days (range, 6–89), and patients were in the intensive care unit for 6 days on average (range, 0–74). Patients were mechanically ventilated for 2.7 days postoperatively (range, 0–62) (9).

Thoracic wall resection and reconstruction are associated with significant morbidity reducing nearly all daily life activities (9). However, a certain degree of selection bias in such assessments must be acknowledged. The patients interviewed here represented the healthier and more active patients. These patients sensed postoperative restrictions more than those patients who were treated in palliative intention because of pain and ulcerated lesions. However, with respect to the malignancy of the underlying disease, these restrictions might be justified. In our patient population, the majority of the treated and interviewed patients would undergo the procedure again (9).

Effective treatment modalities have improved the survival of patients with thoracic wall tumors in recent decades (61). At our institute, the 5-year overall survival rate for patients with malignant chest wall tumors including soft-tissue sarcomas and breast carcinoma was approximately 56% (9). For chest wall sarcomas, the 5-year overall survival rates were approximately 52%. Other studies have presented similar overall survival rates (7, 14, 62, 63). In an analysis of 127 full-thickness resections for chest wall sarcomas, Wouters et al. demonstrated that full-thickness chest wall resection represents a safe and effective procedure, with a limited number of complications and an adequate long-term survival. For primary chest wall sarcomas, these authors reported a 5-year survival rate of 63% and for recurrent sarcomas 50% (14). Furthermore, adjuvant radiotherapy was associated with increased local disease control. In the treatment of locally advanced or recurrent breast carcinomas, full-thickness chest wall resection was also associated with an acceptable morbidity and a 5-year overall survival rate of 63% after surgery (64).

## Conclusion

In curative treatment regimens, chest wall reconstruction enables the complete resection of tumors and subsequent adjuvant radiotherapy. Even at advanced localized disease stages or in a palliative treatment setting, safe and reliable techniques allow the removal of large ulcerative tumors. As a reconstructive option after tumor resection, local flaps represent a reliable tool that can cover most thoracic wall defects. Nevertheless, concerns over distant iatrogenic implantation of tumor cells at the donor site of local flaps do exist when tumor resection and flap coverage are performed during the same surgery. In the literature, unfortunately, there has been no systematic analysis of this issue. However, iatrogenic tumor metastases, especially sarcoma metastases, at donor-tissue sites after local flap reconstruction are a rare occurrence and should not preclude the use of local flap reconstruction (65). They have been reported only in selected case reports (66). Further, the effect of donor-site radiation on the incidence of iatrogenic sarcoma metastases still remains unclear and should be examined (65).

Besides the aforementioned disadvantages and concerns, local flaps can offer some slight but noteworthy advantages. In contrast to free flaps, local flaps do not require intensive postoperative flap inspections. Postoperative positioning protocols and anticoagulation regimens are less stringent. However, there has been a paradigm shift in recent decades. Free tissue transfers can now be performed with a similar or even higher degree of safety than local flap transfer as a result of the improvements in microsurgical techniques. Safe dissection and positioning of a local flap at the chest wall can be technically more demanding, risky, and time-consuming when compared with a free flap transfer in a two-team approach. Due to the microsurgical and anesthesiological improvements, free flap transfers have become physically less demanding surgical procedures and have also become suitable for patients with chest wall tumors at an advanced disease stage.

For those defects that can be covered by local flaps, pectoralis major, thoracoepigastric, VRAM, cranially pedicled TRAM, and latissimus dorsi flaps are commonly used. Small defects can be covered by pectoralis or thoracoepigastric flaps, whereas larger vertical defects can be covered by VRAM flaps. However, the relatively high rates of abdominal hernia have to be considered here. To cover larger defects particularly at the anterolateral thorax, the cranially pedicled TRAM flap can be utilized. However, most of the chest wall defects can be safely covered by pedicled latissimus dorsi flaps, particularly at the anterior chest wall and the sternal region. Pedicled omentum flaps remain an option for large defects with small volumes or when other procedures are unsuitable. Although most chest wall defects can be covered with local flaps, free tissue reconstruction should be carefully considered in each case, especially in areas that are difficult to reach with local flaps or when a single local flap is inadequate to cover the defect. Full-thickness chest wall defects that involve more than four ribs at the lateral chest wall should additionally be stabilized with polypropylene mesh. Larger defects at the anterior and lateral chest wall can be reconstructed by polypropylene mesh/methylmethacrylate composite and covered by local or free flaps.

Conclusively, thoracic wall resection with defect coverage during the same procedure enables patients to recover more quickly and shortens hospitalization. The effects on pulmonary function are moderate and well tolerated. Thoracic wall resection and reconstruction have proven to be safe and effective, with a reasonable long-term survival in the treatment of chest wall sarcomas and locally advanced breast carcinomas. A multimodal approach with proper preoperative evaluation and advanced plastic surgery techniques can decrease postoperative morbidity and ameliorate the resulting functional deficits. Although oncological safety is of the upmost priority, patients’ safety and their quality of life are essential to provide optimal care. Due to the increasing complexity of oncological care and the multiple disciplines involved, this can preferably be achieved in an interdisciplinary approach involving tumor boards at specialized treatment centers.

## Conflict of Interest Statement

The authors declare that the research was conducted in the absence of any commercial or financial relationships that could be construed as a potential conflict of interest.
